# Etiology and antimicrobial resistance of secondary bacterial infections in patients hospitalized with COVID-19 in Wuhan, China: a retrospective analysis

**DOI:** 10.1186/s13756-020-00819-1

**Published:** 2020-09-22

**Authors:** Jie Li, Junwei Wang, Yi Yang, Peishan Cai, Jingchao Cao, Xuefeng Cai, Yu Zhang

**Affiliations:** Department of Pharmacy, Union Hospital, Tongji Medical College, Huazhong University of Science and Technology, Wuhan, 430022 China

**Keywords:** COVID-19, Secondary bacterial infections, Etiology, Antimicrobial resistance, Retrospective analysis

## Abstract

**Background:**

A considerable proportion of patients hospitalized with coronavirus disease 2019 (COVID-19) acquired secondary bacterial infections (SBIs). The etiology and antimicrobial resistance of bacteria were reported and used to provide a theoretical basis for appropriate infection therapy.

**Methods:**

This retrospective study reviewed electronic medical records of all the patients hospitalized with COVID-19 in the Wuhan Union Hospital between January 27 and March 17, 2020. According to the inclusion and exclusion criteria, patients who acquired SBIs were enrolled. Demographic, clinical course, etiology, and antimicrobial resistance data of the SBIs were collected. Outcomes were also compared between patients who were classified as severe and critical on admission.

**Results:**

Among 1495 patients hospitalized with COVID-19, 102 (6.8%) patients had acquired SBIs, and almost half of them (49.0%, 50/102) died during hospitalization. Compared with severe patients, critical patients had a higher chance of SBIs. Among the 159 strains of bacteria isolated from the SBIs, 136 strains (85.5%) were Gram-negative bacteria. The top three bacteria of SBIs were *A. baumannii* (35.8%, 57/159), *K. pneumoniae* (30.8%, 49/159), and *S. maltophilia* (6.3%, 10/159). The isolation rates of carbapenem-resistant *A. baumannii* and *K. pneumoniae* were 91.2 and 75.5%, respectively. Meticillin resistance was present in 100% of *Staphylococcus aureus* and *Coagulase negative staphylococci*, and vancomycin resistance was not found.

**Conclusions:**

SBIs may occur in patients hospitalized with COVID-19 and lead to high mortality. The incidence of SBIs was associated with the severity of illness on admission. Gram-negative bacteria, especially *A. baumannii* and *K. pneumoniae,* were the main bacteria, and the resistance rates of the major isolated bacteria were generally high. This was a single-center study; thus, our results should be externally examined when applied in other institutions.

## Introduction

The severe acute respiratory syndrome coronavirus 2 (SARS-CoV-2), which first appeared in 2019, spread to most of the countries around the world, and the corona virus disease 2019 (COVID-19) has progressed into a global pandemic. Globally, as of July 15, 2020, there have been more than 12 million confirmed cases of COVID-19, including over 570 thousand deaths [1]. According to the previous studies [2, 3], secondary bacterial infection (SBI), which occurs at an approximate incidence of 10% ~ 15%, is a dangerous and common complication in patients hospitalized with COVID-19. According to existing reports, 50% of COVID-19 deaths experienced secondary bacterial infections (SBIs); thus, patients with SBIs have a higher risk of mortality [3, 4]. SBIs had become the hidden threat lurking behind COVID-19. The effective antimicrobial regimen is still one of the key measures for the successful treatment of COVID-19 [5].

Due to the lack of controlled clinical trials about the use of empiric antibacterial agents in COVID-19 patients, the current recommendations are based upon extrapolation of data from other viral pneumonia [5]. A quick guide [6] has recommended empiric antimicrobial treatment for all possible bacteria in severe COVID-19 patients with SBIs. Also, empiric use of third-generation cephalosporin combined enzyme inhibitor for SBIs has been recommended in severe patients [7]. Yet, the SBIs caused by COVID-19 tend to differ from other forms of SBIs. During the outbreak, a large number of broad-spectrum antibacterial agents were used, and the vast majority of patients hospitalized with COVID-19 were given empirical antimicrobial treatment before SBIs were confirmed [2, 3, 8]. The broader application of antibacterial agents may further lead to changes in etiology and antimicrobial resistance. The SBIs in patients hospitalized with COVID-19 should be treated according to further microbiological data. Currently, there is no report on the pathogenic spectrum of SBIs. Some cases of bacterial infections have been reported in the research about the clinical characteristics of COVID-19; however, these were no systematic studies on the etiology of SBIs, and the number of positive cultures was small [8–12]. Merely indicating the distribution of bacteria is not enough to guide reasonable empiric use of antibacterial agents.

Consequently, in the present study, we conducted a first large sample size retrospective analysis of SBIs in patients hospitalized with COVID-19. The aim was to obtain the etiology and antimicrobial resistance of SBIs for more accurate antimicrobial use.

## Materials and methods

### Study population

This single-center, retrospective study was done at Wuhan Union Hospital, which was a designated hospital to treat patients with COVID-19 in Wuhan, China. A total of 1495 patients were diagnosed as COVID-19 and treated in the West Campus of Wuhan Union Hospital between January 27 and March 17, 2020. According to the severity of illness on admission, 1050 of them were classified as severe (i.e., dyspnea, respiratory frequency ≤ 30/min, blood oxygen saturation ≤ 93%, the partial pressure of arterial oxygen to fraction of inspired oxygen ratio < 300, and/or lung infiltrates > 50% within 24 to 48 h) and 258 patients were critical (i.e., respiratory failure, septic shock, and/or multiple organ dysfunction or failure). Demographic, clinical course, laboratory, and treatment data were collected from electronic medical records.

### Study design

SBIs were defined when patients showed clinical characteristics of bacterial infections, and at least one positive etiology of bacteria was acquired from qualified microbiological specimens (qualified sputum, endotracheal aspirate, bronchoalveolar lavage fluid, blood samples, or qualified urine) after SARS-CoV-2 infection [3, 13]. We performed a retrospective review of medical records that met the criteria from January 27 to March 17, 2020. Inclusion criteria were: (1) patients diagnosed with COVID-19 according to the Guidance for COVID-19 (7th edition) released by the National Health Commission of China [14]; (2) met the diagnostic criteria of SBIs. Patients were excluded if: (1) before being infected with SARS-CoV-2, they had other infectious diseases; (2) the medical records were incomplete. Patients enrolled in the study were basically severe or critically ill. Therefore, according to the severity of illness on admission, the enrolled patients were divided into severe group and critical group.

The study was approved by the Ethics Committee of Union Hospital, Tongji Medical College, Huazhong University of Science and Technology (Permission number: [2020]0104).

### Pathogen detection and antimicrobial susceptibility

The qualified microbiological specimens of patients with COVID-19 from January 27, 2020 to March 17, 2020 were collected and cultured. Pathogen identification and antimicrobial susceptibility testing were carried out on the Phoenix-100 automatic microbiological system (BD Corporation, USA). In some further antimicrobial susceptibility testing, the international Kirby-Bauer method was also used. All the results were interpreted according to the criteria of the Clinical and Laboratory Standards Institute (CLSI 2019) [15]. The same strains from one patient were counted only once. The data were analyzed using WHONET 5.6 software (World Health Organization).

### Statistical analysis

Continuous and categorical variables were presented as median (IQR) and percentages. We assessed differences between the severe and critical groups using the Mann-Whitney U test for continuous variables and χ^2^ test, or Fisher’s exact test for categorical variables. A *P*-value < 0.05 was regarded as statistically significant. All statistical analyses were performed by IBM SPSS Statistics 26.0.

## Results

### General information

After excluding 9 patients who had other infectious diseases before being infected with SARS-CoV-2, and 7 in patients with incomplete medical records, a total of 102 patients (6.8%, 102/1495) were included in the study. The mean age was 66.2 ± 11.2 years (30 ~ 93 years; Table 1), and 68 patients (66.7%) were males. Compared with the severe group, the critical group was more likely to acquire SBIs (69/258 [26.7%] vs. 33/1050 [3.1%]). Almost half of the patients who acquired SBIs (49.0%, 50/102) died during hospitalization, and the other patients were discharged. Compared with the severe group, the critical group had a significantly increased mortality (45/69 [65.2%] vs. 5/33 [15.2%], *P* < 0.0001).

**Table 1:** Demographic, clinical course and outcome data of patients who acquired SBIs during the COVID-19 hospitalization

	All patients (*n* = 102)	Severe group (*n* = 33)	Critical group (*n* = 69)	*P*-value
Characteristics				
Age, years	66.2 (30 ~ 93)	64.9 (30 ~ 82)	66.1 (36 ~ 93)	0.686
Sex				0.178
Men	68 (66.7%)	19 (57.6%)	49 (71.0%)	
Women	34 (33.3%)	14 (42.4%)	20 (29.0%)	
Bacterial etiology				
*A. baumannii*^a^	50 (49.0%)	10 (30.3%)	40 (58.0%)	0.009
*CRAB*^a^	47 (46.1%)	9 (27.3%)	38 (55.1%)	0.008
*K. pneumoniae*^a^	35 (34.3%)	6 (18.2%)	29 (42.0%)	0.018
*CRKP*^a^	32 (31.4%)	5 (15.2%)	27 (39.1%)	0.015
Treatment before SBIs				
Antiviral therapy	96 (94.1%)	29 (87.9%)	67 (97.1%)	0.084
Antibiotic therapy	99 (97.1%)	31 (93.9%)	68 (98.6%)	0.244
Outcomes				< 0.0001
Discharge	52 (51.0%)	28 (84.8%)	24 (34.8%)	
Death	50 (49.0%)	5 (15.2%)	45 (65.2%)	

Data are median (IQR) or n (%). *P* values comparing severe group and critical group are from Mann-Whitney U test, χ^2^ test, or Fisher’s exact test

^a^Number of patients suffering from a certain bacterial infection

The proportion of SBIs in the lungs, bloodstream, and urinary tract was 86.3% (88/102), 34.3% (35/102), and 7.8% (8/102), respectively. Moreover, 27 (26.5%) patients had lung infections mixed with bloodstream infections; 2 (2.0%) patients had urinary tract infections. There was no secondary infection in other sites.

### Etiology of the secondary infection

A total of 159 strains of bacteria were isolated from the cultures in the 102 patients. Among the isolated bacteria, Gram-negative bacteria were the main bacteria, accounting for 85.5%. The top three bacteria of SBIs were *Acinetobacter baumannii* (*A. baumannii*, 35.8%), *Klebsiella pneumoniae* (*K. pneumoniae*, 30.8%), and *Stenotrophomonas maltophilia* (*S. maltophilia*, 6.3%). The distribution and composition ratios of bacteria are shown in Table 2. Among them, 46 patients had infections with mixed bacteria, mostly *A. baumannii* mixed with *K. pneumoniae* (41.3%)(Table 3).

**Table 2:** Etiological distribution of SBIs in patients hospitalized with COVID-19

Bacteria	N (%) in different sites			
	Lungs	bloodstream	Urinary tract	Total
Gram-negative	105 (95.5)	27 (62.8)	4 (50.0)	136 (85.5)
*A. baumannii*	47 (42.7)	9 (20.9)	1 (12.5)	57 (35.8)
*K. pneumoniae*	34 (30.9)	15 (34.9)	0 (0)	49 (30.8)
*S. maltophilia*	10 (9.1)	0 (0)	0 (0)	10 (6.3)
*Pseudomonas aeruginosa*	7 (6.4)	0 (0)	0 (0)	7 (4.4)
*Escherichia coli*	4 (3.6)	1 (2.3)	3 (37.5)	8 (5.0)
others	3 (2.7)	2 (4.7)	0 (0)	5 (3.1)
Gram-positive	5 (4.5)	16 (37.2)	2 (25.0)	23 (14.5)
*Staphylococcus aureus*	2 (1.8)	1 (2.3)	0 (0)	3 (1.9)
*Staphylococcus epidermidis*	0 (0)	2 (4.7)	0 (0)	2 (1.3)
*Staphylococcus hominis*	0 (0)	5 (11.6)	0 (0)	5 (3.1)
*Staphylococcus haemolyticus*	0 (0)	2 (4.7)	0 (0)	2 (1.3)
*Enterococcus faecium*	0 (0)	4 (9.3)	2 (25.0)	6 (3.8)
others	3 (2.7)	2 (4.7)	0 (0)	5 (3.1)
Total N (%)	110 (100)	43 (100)	6 (100)	159 (100)

**Table 3:** Etiological distribution of SBIs caused by multiple bacteria in patients hospitalized with COVID-19

Mixed infection	N (%)
Two bacteria	
*A. baumannii + K. pneumoniae*	9 (19.6)
*A. baumannii + staphylococcus*	4 (8.7)
Other combination	17 (37.0)
Three and more bacteria	16 (34.8)
Total N (%)	46 (100)

### Antimicrobial susceptibility

The antimicrobial resistance rate of bacteria isolated from patients with SBIs was generally high. The isolation rates of carbapenem-resistant *A. baumannii* (CRAB) and carbapenem-resistant *K. pneumoniae* (CRKP) were 91.7 and 76.6%, respectively. The infection rates of CRAB and CRKP in the critical group were significantly higher than in the severe group (*P* < 0.05). Meticillin resistance was present in 100% of *Staphylococcus aureus* and *Coagulase negative staphylococci*, and vancomycin resistance was not found. The isolation rate of extended-spectrum beta-lactamase (ESBL)-producing *Escherichia coli* (*E. coli*) was 75%. The results of antimicrobial susceptibility testing for the major bacteria are shown in Table 4 and Table 5.

**Table 4:** Antimicrobial susceptibility of major Gram-negative bacteria

Antibacterial	Major Gram-negative bacteria, N (%) of resistant strains				
	*A. baumannii (n* = 57)	*K. Pneumoniae* (*n* = 49)	*S. Maltophilia* (*n* = 10)	*P.aeruginosa* (*n* = 7)	*E. coli (n* = 8)
Ampicillin	–	49 (100)	–	–	7 (87.5)
Ampicillin sulbactam	53 (93.0)	44 (89.8)	–	–	2 (25.0)
Piperacillin	53 (93.0)	43 (87.8)	–	1 (14.3)	7 (87.5)
Piperacillin tazobactam	52 (91.2)	38 (77.6)	–	1 (14.3)	1 (12.5)
Amoxicillin clavulanate	57 (100)	42 (85.7)	–	–	1 (12.5)
Cefazolin	–	48 (98.0)	–	–	7 (87.5)
Cefuroxime	–	43 (87.8)	–	–	6 (75.0)
Ceftriaxone	52 (91.2)	41 (83.7)	–	–	6 (75.0)
Ceftazidime	52 (91.2)	41 (83.7)	9 (90.0)	1 (14.3)	3 (37.5)
Cefoperazone sulbactam	45 (78.9)	39 (79.6)	–	–	–
Cefepime	53 (93.0)	41 (83.7)	–	1 (14.3)	5 (62.5)
Aztreonam	57 (100)	41 (83.7)	–	2 (28.6)	2 (25.0)
Cefoxitin	–	41 (83.7)	–	–	0 (0)
Meropenem	52 (91.2)	37 (75.5)	–	3 (42.9)	0 (0)
Imipenem	52 (91.2)	37 (75.5)	–	3 (42.9)	0 (0)
Amikacin	48 (84.2)	36 (73.5)	–	0(0)	0 (0)
Gentamicin	52 (91.2)	39 (79.6)	–	0(0)	2 (25.0)
Tobramycin	50 (87.7)	32 (65.3)	–	–	2 (25.0)
Levofloxacin	52 (91.2)	39 (79.6)	3 (30.0)	2 (28.6)	6 (75.0)
Ciprofloxacin	52 (91.2)	43 (87.8)	–	2 (28.6)	6 (75.0)
Sulfamethoxazole trimethoprim	48 (84.2)	36 (73.5)	0 (0)	–	4 (50.0)
Minocycline	16 (28.1)	23 (46.9)	0 (0)	–	2 (25.0)
Tigecycline	0 (0)	1 (1.7)	–	–	0 (0)
ESBL	–	43 (87.8)	–	–	6 (75.0)

Note:- Not detected

**Table 5:** Antimicrobial susceptibility of major Gram-positive bacteria

Antibacterial	Major Gram-positive bacteria, N (%) of resistant strains		
	*Staphylococcus aureus* (*n* = 3)	*Coagulase negative staphylococci* (*n* = 11)	*Enterococcus faecium (n* = 6)
Penicillin G	3 (100)	11 (100)	6 (100)
Oxacillin	2 (66.7)	11 (100)	–
Ampicillin	–	–	6 (100)
Erythromycin	2 (66.7)	11 (100)	6 (100)
Clindamycin	1 (33.3)	6 (54.5)	–
Minocycline	0 (0)	0 (0)	0 (0)
Levofloxacin	–	–	5 (83.3)
Ciprofloxacin	1 (33.3)	10 (90.9)	6 (100)
Gentamicin	1 (66.7)	3 (27.3)	2 (33.3)
Vancomycin	0 (0)	0 (0)	0 (0)
Teicoplanin	0 (0)	0 (0)	0 (0)
Linezolid	0 (0)	0 (0)	0 (0)

Note:- Not detected

## Discussion

Respiratory failure or multiple organ failure is the direct cause of death in patients with COVID-19, and SBIs have an important role in this process [16]. Among the 1495 patients with COVID-19, the incidence of SBIs was 6.8%. The incidence of SBIs was lower than the data in previous studies (10% ~ 15%, Wuhan, China), which may be due to the larger sample size in the present study [2, 3]. In the mild ill COVID-19 patients, there was no SBI that met the inclusion and exclusion criteria; thus, it was impossible to compare the differences between the mild group and the severe group. The incidence in the critical group was much higher than in the severe group, which was consistent with the higher rate of central catheter placement and invasive mechanical ventilation in critical patients [2]. Almost half (49.0%) of the patients with SBIs died during hospitalization, which was consistent with the previous study (50%) [3]. Compared with the severe group, the critical group had significantly increased mortality. Recent studies related to COVID-19 reported that the male gender was a risk factor for disease severity status, and age 65 or older was a risk factor related to death [3, 17, 18]. In our research, no differences in gender and age were found between the severe and critical groups, which suggested that gender and age were not risk factors for death in patients with SBIs. *A. baumannii* and *K. pneumoniae* were the main pathogens of SBIs, and the infection rates of *A. baumannii*, CRAB, *K. pneumoniae* and CRKP in critical group were significantly higher than in the severe group. As the mortality of CRAB and CRKP has always been high, we believe it is one of the reasons why the mortality rate in the critical group was higher than that in the severe group.

According to the sites of SBIs, lung infections were the main type, which may be related to the decrease of airway defense function after SARS-CoV-2 infection [19]. Invasive operations such as trachea intubation and ventilator-assisted breathing during hospitalization may also be the causes of SBIs in the lungs. There were 35 patients with bloodstream infections, 27 of which were bloodstream infections mixed with lung infections. We compared the bacteria of mixed infections and found that 21 patients had the same bacteria in the lungs and bloodstream, including *K. pneumoniae* (66.7%, 14/21) and *A. baumannii* (33.3%, 7/21). In these 21 patients, lung infections occurred first, followed by bloodstream infections. The antibiogram reportings of *K. pneumoniae* and *A. baumannii* isolated from qualified sputum specimens and blood specimens were the same. It is possible that the migration of *K. pneumoniae* or *A. baumannii* from the lungs resulted in bloodstream infections in these patients.

A total of 159 strains of bacteria isolated in this study were mainly Gram-negative bacteria. The top three bacteria of secondary lung infections were *A. baumannii*, *K. pneumoniae*, and *S. maltophilia*. The etiological distribution was different from the previously reported bacteria of hospital-acquired pneumonia (HAP) [20, 21]. The proportion of *A. baumannii and K. pneumoniae* was significantly increased, and the proportion of *Pseudomonas aeruginosa* (*P. aeruginosa*) and *Staphylococcus aureus* (*S. aureus*) was decreased, which suggested that the initial empirical antimicrobial program of HAP should not be completely copied if SBIs occur in the lungs. The lower proportion of *P. aeruginosa* and *S. aureus* suggests that it is not necessary to first choose antimicrobial with antibacterial activity of *P. aeruginosa* and *S. aureus* for SBIs in the lungs*.* The choice of antimicrobial program could be more suitable to treat the infections of *A. baumannii and K. pneumoniae.* The antimicrobial susceptibility tests showed that most of *A. baumannii and K. pneumoniae* were multi-drug resistant bacteria. The isolation rates of CRAB and CRKP were 91.7 and 76.6%, respectively. When patients suffer from SBIs, the possibility of infections by drug-resistant strains should be adequately considered. The resistance rate of tigecycline and cefoperazone sulbactam was relatively lower, and the combination could be considered for the initial empirical treatment of SBIs in the lungs. According to reports [22, 23], the avibactam compound has a better effect on carbapenem-resistant *K. pneumoniae*; yet, there is still no systematic research in patients with COVID-19.

Although the bacteria of secondary bloodstream infections were mainly Gram-negative bacteria, the proportion of Gram-positive bacteria was relatively higher than lung infections. If the bacteria derived from lung infections were excluded from the statistics, Gram-positive bacteria would be the main bacteria for bloodstream infections. In this study, we found that 80.0% (16/20) of patients infected with Gram-positive bacteria were given central venous catheter implantation during hospitalization. Our results revealed that the bloodstream infections of Gram-positive bacteria were associated with central venous catheter implantation. Therefore, we suggest that the management of venous catheters in severe patients should be strengthened to avoid bloodstream infections. According to antimicrobial susceptibility tests, methicillin resistance was found in 100% of *Staphylococcus aureus* and *Coagulase negative staphylococci*, and vancomycin resistance was not yet found. This suggests that vancomycin can be used as the empirical choice for Gram-positive bacteria if secondary bloodstream infections occur.

The number of secondary urinary tract infections was relatively small, and *E. coli* was still the main bacterium. According to antimicrobial susceptibility tests, the isolation rate of ESBL-producing *E. coli* was 75%. As the initial empirical choice, β-lactams combinations with β-lactamase inhibitors could be recommended, rather than levofloxacin and ceftriaxone.

This study has several limitations. First, this was a single-center study performed in the Wuhan Union Hospital. The etiology and antimicrobial resistance in different medical institutions or different regions may be different. The results should be externally examined when applied in other institutions. Second, during the epidemic, the main focus was dedicated to treating COVID-19 patients; thus, there was no enough time to examine the mechanism of bacterial resistance. Third, our analysis of the treatment effect of SBIs was insufficient, which should be carried out in further research.

## Conclusions

SBI is one of the main complications in patients hospitalized with COVID-19 that leads to high mortality. Gram-negative bacteria, especially *A. baumannii* and *K. pneumoniae*, are the main bacteria. The antimicrobial resistance rates of the major isolated bacteria are generally high, which indicates that more accurate use of antibacterial agents is necessary for SBIs in patients hospitalized with COVID-19.

## Data Availability

The supporting data are available from the corresponding author and laboratory depositories.

## Abbreviations

COVID-19: Corona virus disease 2019
SBIs: Secondary bacterial infections
HAP: Hospital-acquired pneumonia
SARS-CoV-2: Severe acute respiratory syndrome coronavirus 2
CLSI: Clinical and Laboratory Standards Institute
*A. baumannii*: *Acinetobacter baumannii*
*K. pneumoniae*: *Klebsiella pneumoniae*
*E. coli*: *Escherichia coli*
*S. maltophilia*: *Stenotrophomonas maltophilia*
*P. aeruginosa*: *Pseudomonas aeruginosa*
*S. aureus*: *Staphylococcus aureus*
CRAB: Carbapenem-resistant *A. baumannii*
CRKP: Carbapenem-resistant *K. pneumoniae*
